# Body Posture and Low Back Pain in Amateur Tennis Players: A Cross-Sectional Observational Study

**DOI:** 10.3390/jcm15114000

**Published:** 2026-05-22

**Authors:** Izabela Rutkowska, Jakub Olewiński, Paweł Tomaszewski, Wiktoria Bandura

**Affiliations:** 1 Department of Movement Teaching, Faculty of Rehabilitation, Józef Piłsudski University of Physical Education in Warsaw, 00-968 Warsaw, Poland; izabela.rutkowska@awf.edu.pl (I.R.); kuba.olewinski@wp.pl (J.O.); 2 Department of Tourism and Recreation, Faculty of Physical Education, Józef Piłsudski University of Physical Education in Warsaw, 00-968 Warsaw, Poland

**Keywords:** tennis, low back pain, body posture, Diers Formetric

## Abstract

**Background**: Quantitative assessment of postural parameters in all anatomical planes may help clarify the potential role of posture in the pathomechanism of low back pain (LBP) in tennis players and support the development of preventive and rehabilitation strategies. This observational cross-sectional study aimed to compare selected pelvic and spinal posture parameters of amateur tennis players with LBP, amateur tennis players without LBP, and physically active controls without LBP who did not participate in asymmetric sports. **Methods**: The study included 116 participants (52 women and 64 men): amateur tennis players with self-reported tennis-limiting LBP within the previous 6 months, amateur tennis players without such a history, and physically active controls who did not participate in asymmetric sports and were free of LBP. Spinal and pelvic posture parameters were assessed using the DIERS Formetric 4D rasterstereography system. Group comparisons were performed using ANOVA or the Kruskal–Wallis test, as appropriate. **Results**: Across most analysed body posture parameters, no clear and consistent between-group differences were detected in this sample. Among men, significant between-group differences were observed in pelvic rotation and maximum right vertebral rotation. Men in the TBP (Tennis Back Pain) group showed a mean left-sided pelvic rotation compared with a mean right-sided rotation in those in the TNBP (Tennis No Back Pain) group, and lower maximum right vertebral rotation than men in the PAC (Physically Active Control) group. Among women, a significant between-group difference was observed for coronal imbalance, with higher left-sided values in the TBP group than in PACs. No consistent between-group differences were found across the remaining pelvic and spinal parameters. **Conclusions**: In this sample of amateur tennis players, static body posture parameters showed limited and non-uniform associations with LBP. The observed differences were selective and of uncertain clinical relevance, and the analyses did not reveal a single consistent postural pattern clearly associated with LBP in this sample.

## 1. Introduction

Tennis is one of the most commonly practiced individual sports worldwide, and regular amateur participation provides measurable health benefits, including improvements in aerobic fitness and body composition [1,2]. However, playing tennis is associated with a substantial risk of musculoskeletal injuries, particularly low back pain (LBP) [3]. According to Abrams et al. [4], the prevalence of LBP among tennis players may be as high as 85%. In this population, LBP not only results in absence from training sessions and tournaments but also reduces quality of life and limits physical activity.

The etiology of LBP in tennis players is complex and multifactorial. The structures of the spine and hip joints play a crucial role in transmitting force between the lower limbs and the racket [5]. Combined with high movement velocity, repetitive loading [6], and the sport’s asymmetrical nature, these factors lead to considerable mechanical stress on the lumbar spine [7]. Repetitive multiplanar loading generates substantial forces that act on spinal structures—including intervertebral discs, facet joints, and ligaments—predisposing them to overload and microtrauma [8].

In addition to external factors such as stroke technique [9] and training load [10], numerous internal predispositions contributing to LBP have been identified. These commonly include trunk asymmetry, imbalance of spinal stabilizing muscles, restricted lumbopelvic mobility, and abnormalities in spinal and pelvic alignment [11,12]. A meta-analysis by Sugavanam et al. [13] demonstrated an association between anterior pelvic tilt and chronic LBP, although findings regarding lordotic and kyphotic curvatures remain inconsistent. While some studies suggest a possible relationship between selected postural parameters and LBP [14,15,16], others do not confirm such an association [17,18,19].

Currently, the standard for safe and precise assessment of static body posture is three-dimensional surface imaging techniques. One such tool is the Diers Formetric 4D system, which is based on raster stereography [20,21]. This system enables three-dimensional reconstruction of spinal shape and pelvic alignment by analyzing back-surface topography without ionizing radiation, allowing for repeated measurements without radiation exposure [20]. Numerous studies have confirmed the reliability and reproducibility of measurements obtained with this method [20,21,22]. Rasterstereography has also proven useful in detecting subtle postural deviations associated with spinal pain. Schroeder et al. [23] reported significant differences in spinal alignment between patients with chronic LBP and healthy individuals, as measured by back-surface topography analysis. The application of modern 3D posture imaging techniques in research on tennis players may therefore provide new insights into potential associations between characteristic postural features and the occurrence of LBP.

Most research involving tennis players has focused on biomechanical analysis of strokes, particularly the serve. Functional factors such as trunk muscle strength and endurance, as well as range of motion, have also been investigated in relation to LBP [12,24]. However, a research gap remains regarding the influence of long-term amateur tennis participation on specific alterations in spinal and pelvic alignment. Studies addressing similar issues have been conducted among handball players [25], table tennis players [26], and volleyball players [27], yet no comparisons have been made between tennis players with and without LBP relative to individuals who do not practice asymmetrical sports.

Analysis of body posture in tennis players and in physically active individuals who do not play tennis may reveal potential postural compensations associated with participation in an asymmetrical sport. Quantitative assessment of static postural parameters in all anatomical planes may help clarify whether relaxed standing posture is associated with LBP in tennis players; however, such an assessment does not capture dynamic spinal loading or sport-specific biomechanics.

Therefore, this observational cross-sectional study aimed to compare selected pelvic and spinal posture parameters of amateur tennis players with LBP, amateur tennis players without LBP, and physically active controls without LBP who did not participate in asymmetric sports. The analysis was exploratory in nature, particularly with respect to sex-stratified comparisons, and the study was designed to assess posture parameters in relation to a recent history of tennis-limiting low back pain rather than clinically subclassified low back pain entities.

## 2. Materials and Methods

### 2.1. Participants

This was an observational cross-sectional study involving 116 participants (52 women and 64 men), comprising amateur tennis players and individuals who did not play tennis but engaged in other physical activities (running, swimming, cycling, fitness, or gym-based exercise). The study was conducted in accordance with the Declaration of Helsinki at the Mirai Rehabilitation Institute in Warsaw between 1 April 2022 and 30 November 2022. Approval for the study was obtained from the Senate Committee on Research Ethics (SKE 01-53/2021), and the study was registered in the ClinicalTrials.gov (Protocol Registration and Results System) as an observational study (Identifier: NCT06890845, registration date: 24 March 2025). All participants provided written informed consent prior to the study and were informed that they could withdraw from the research at any stage without providing a reason. The study is reported in accordance with the STROBE statement for cross-sectional studies.

The following inclusion criteria were applied:

Tennis back pain (TBP) group (n = 41, M = 26, F = 15): (a) age 18–50 years; (b) tennis practice at least 3 times per week for the past 2 years; (c) written informed consent; and (d) self-reported low back pain within the previous 6 months that was severe enough to limit tennis participation. No specialist-confirmed clinical diagnosis or pathoanatomical subclassification was required for group allocation.

Tennis no back pain (TNBP) group (n = 48, M = 23, F = 25): (a) age 18–50 years; (b) tennis practice at least 3 times per week for the past 2 years; (c) written informed consent; and (d) no episode of low back pain within the previous 6 months, during which, there was limited tennis participation.

Physically active controls (PACs) (n = 27, M = 15, F = 12): (a) age 18–50 years; (b) written informed consent; (c) no episode of low back pain within the previous 6 months; and (d) engagement in physical activity other than tennis (running, swimming, cycling, fitness, or gym-based exercise) at least 3 times per week for the past 2 years. This group was intended to provide an active comparison sample but was not formally matched to the tennis groups in terms of total training load or type of physical activity.

Exclusion criteria for all groups included: (a) lack of written consent; (b) history of spinal or trunk surgery; (c) primary musculoskeletal conditions, e.g., previous trunk neoplasms, vertebral fractures, or structural scoliosis; (d) pain present on the day of assessment. This approach was intended to reduce the influence of acute pain-avoidance strategies on standing posture; however, it also means that the assessment may not fully reflect posture adaptations present during symptomatic states.

Eligibility criteria related to tennis participation, general physical activity, and low back pain history were based on participants’ self-reports obtained during screening.

Detailed demographic and clinical characteristics of the study groups are presented in the Results section (Table 1). The final sample comprised 116 participants, exceeding the a priori estimated minimum sample size for the planned between-group comparisons.

### 2.2. Procedures

Participants were recruited through announcements posted in tennis clubs, on online platforms, and in social media groups where amateur tennis players gather. Individuals interested in participating received electronic documentation describing the study procedures and a written informed consent form. Initial eligibility screening was then conducted by telephone to verify compliance with the inclusion and exclusion criteria. The information obtained during screening, including tennis participation frequency, duration of physical activity, and history of low back pain, was based on self-reports and was not verified against training logs, club records, medical records, or other external objective sources.

**Figure 1:**
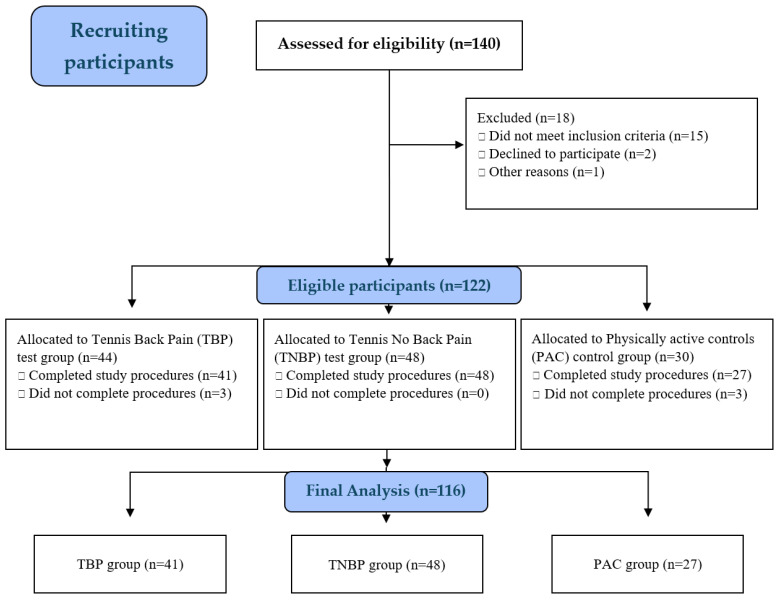
Flow diagram of participant recruitment and selection.

In total, 140 individuals were assessed for eligibility. Eighteen were excluded: 15 did not meet the inclusion criteria, 2 declined to participate, and 1 was excluded for other reasons. Of the remaining participants, 44 were classified as the tennis back pain (TBP) group, 48 as the tennis no back pain (TNBP) group, and 30 as the physically active control (PAC) group. The study procedures were completed by 41, 48, and 27 participants, respectively, and all of them were included in the final analysis (Figure 1).

Spinal posture was assessed using the Formetric 4D rasterstereography system (DIERS International GmbH, Schlangenbad, Germany). This method is well established and reliable for three-dimensional analysis of spinal alignment [1,2]. Before the assessment, all participants were provided with detailed information regarding the measurement procedures and study methodology. It was assumed that individuals experiencing LBP during assessment could adopt a non-habitual posture, potentially resulting in an unnatural positioning of body segments.

Therefore, participants were asked whether they were experiencing pain at the time of measurement. None of the participants reported pain during the examination. Participants were instructed to remove all clothing except short sports shorts. The back was exposed from the gluteal cleft to the occipital region. Participants stood barefoot in a comfortable upright posture, with knees extended and the upper limbs hanging freely alongside the trunk. To standardize body positioning, all participants stood at the exact marked location on the measurement system’s treadmill platform. Participants were positioned 2 m from the Formetric 4D projection and camera unit. The projection unit emitted structured-light stripes onto the participant’s back. In accordance with the recommendations of Guidetti et al. [28], no reflective markers were applied to the body. Each scan was performed using DIERS acquisition and processing software. Twelve images were recorded within 6 s for each measurement (2 Hz). All scans were processed according to the manufacturer’s guidelines. On each captured frame, the software automatically detected the left and right sacral dimples (DL/DR), corresponding to the posterior superior iliac spines, and the spinous process of vertebra C7 (VP). The midpoint between the dimples (DM) was calculated based on the locations of DL and DR.

The primary variables of interest were selected pelvic and spinal posture parameters obtained from rasterstereographic assessment using the DIERS Formetric 4D system.

Participants were examined individually and received identical instructions before the assessment. The following parameters were evaluated:

(a) Lumbar lordosis angle (°): angle between the tangential surfaces at the thoracolumbar and lumbosacral transition points.
(b) Thoracic kyphosis angle (°): angle between the tangential surfaces at the cervicothoracic and thoracolumbar transition points.
(c) Fleché lombaire (mm): distance between the apex of lumbar lordosis and the tangent drawn through the apex of thoracic kyphosis.
(d) Fleché cervicale (mm): distance between the apex of cervical lordosis and the tangent drawn through the apex of thoracic kyphosis.
(e) Pelvic tilt (mm): difference in vertical height between the right and left sacral dimples.
(f) Pelvic torsion (°): angle between normals (perpendiculars to the local tangent plane) at the right and left sacral dimples.
(g) Pelvic rotation (°): frontal plane rotation of the right sacral dimple relative to the left sacral dimple.
(h) Apical deviation +max (maximum deviation to the right): the greatest lateral deviation of the spinous process line from the line connecting C7 and the midpoint between the sacral dimples (mm)
(i) Apical deviation -max (maximum deviation to the left): the greatest lateral deviation of the spinous process line from the line connecting C7 and the midpoint between the sacral dimples (mm)
(j) Apical deviation RMS (mm): root mean square of the deviation of the spinal midline from the segment connecting C7 to the midpoint between the sacral dimples.
(k) Trunk torsion (°): maximum horizontal component at the C7 level compared with that of the midline between the sacral dimples.
(l) Vertebral rotation RMS (°): root mean square of horizontal components of surface normals along the spinal midline.
(m) Maximum vertebral rotation to the right (+max) (°): maximal horizontal component of surface normals to the right along the spinal midline.
(n) Maximum vertebral rotation to the left (−max) (°): maximal horizontal component of surface normals to the left along the spinal midline.
(o) Coronal imbalance (mm): the horizontal distance between the vertebral prominence (VP, C7) and the midpoint between the sacral dimples (DM), reflecting lateral trunk displacement relative to the pelvis in the frontal plane.
(p) Scoliotic angle: the angle representing the magnitude of lateral spinal curvature in the frontal plane, calculated from the reconstructed spinal midline based on surface topography.

To minimize potential bias, several measures were taken. To reduce selection-related bias, strict inclusion and exclusion criteria were applied and checked during telephone screening; however, the corresponding eligibility information was self-reported and not independently verified.

To reduce measurement bias, all postural assessments were performed using the same DIERS Formetric 4D system by a single trained examiner, following the same standardized protocol and under identical testing conditions for all participants. Participants reporting pain on the day of assessment were excluded to ensure that the recorded posture was habitual and not influenced by acute pain-avoidance mechanisms.

### 2.3. Statistical Analysis

Descriptive statistics were used to characterise the analysed variables, including the mean and measures of dispersion, such as the standard deviation, minimum, and maximum. The normality of distribution was assessed using the Shapiro–Wilk test, while homogeneity of variances was evaluated using Levene’s test. For parametric comparisons, analysis of variance (ANOVA) was applied to examine the effects of group (TBP vs. TNBP vs. PAC), sex (M vs. F), and the group × sex interaction. For variables that did not meet the assumptions of parametric tests, differences between groups were analysed using the Kruskal–Wallis test. When significant differences were detected, pairwise comparisons were performed using Dunn’s post hoc test. Post hoc comparisons for ANOVA were conducted using Tukey’s test. Effect sizes were estimated using partial eta squared (η^2^) for ANOVA and epsilon squared (ε^2^) for the Kruskal–Wallis test. The magnitude of effect sizes was interpreted as small (0.01–0.06), moderate (0.06–0.14), and large (≥0.14).

An a priori sample size calculation was performed using G*Power 3 software. The primary analysis was based on models including group and sex as factors. The power calculation referred to the overall between-group comparisons in the full sample and was not intended to ensure adequate statistical power for sex-stratified analyses. Accordingly, sex-specific analyses were treated as exploratory.

With statistical power set at 0.80, α = 0.05, and an assumed effect size of 0.30, the required total sample size for between-group comparisons ranged from 88 participants for the chi-square test to 102 participants for ANOVA, depending on the test used.

Because numerous posture parameters were analyzed and some subgroup comparisons involved relatively small sample sizes, isolated statistically significant findings and non-significant subgroup results were interpreted cautiously. All statistical analyses were performed using STATISTICA 13.0 PL software. The level of statistical significance was set at α = 0.05.

There were no missing data for the variables included in the final analyses; therefore, no data imputation procedures were required.

## 3. Results

The study flow and participant characteristics are presented in Figure 1 and Table 1, respectively. The groups were broadly comparable in terms of age, body mass, height, and BMI, although no formal matching procedure was used.

### 3.1. Body Posture Parameters—Pelvis

Across the analysed pelvic parameters, no clear and consistent between-group differences were detected in this sample. Given the subgroup sample sizes, these findings should be interpreted with appropriate caution.

A significant between-group difference (H(2) = 6.98, *p* = 0.031, ε^2^ = 0.08) was observed in pelvic rotation among men, with the TBP group showing a mean left-sided rotation and the TNBP group showing a mean right-sided rotation. The effect of sex and the group × sex interaction was not statistically significant.

Among women, a statistically significant between-group difference (H(2) = 7.78, *p* = 0.020, ε^2^ = 0.14) was observed for coronal imbalance, with higher left-sided values in the TBP group than in PACs. The effects of sex and the group × sex interaction were not statistically significant.

No statistically significant differences were found for the remaining pelvic parameters (Table 2).

### 3.2. Body Posture Parameters—Spine

Across most spinal posture parameters, the analyses did not reveal clear and consistent between-group differences in this sample. These findings should be interpreted with caution given the subgroup sample sizes.

Statistically significant differences were observed among men in maximum right vertebral rotation (H(2) = 8.05, *p* = 0.018, ε^2^ = 0.09). Men in the TBP group showed a mean maximum right vertebral rotation of 1.27°, which, compared with men in the PAC group (mean 2.87°), was statistically significant (*p* = 0.021; Table 3).

No statistically significant differences were observed between the study groups in lumbar lordosis angle values (*p* > 0.05). Mean values for men in the TBP, TNBP, and PAC groups were 43.8°, 35.9°, and 34.8°, respectively, and for women, they were 46.7°, 48.4°, and 48.2° (Table 3). A statistically significant effect was found for sex (F_2,100_ = 50.14, *p* < 0.001, η^2^ = 0.334), with higher values observed in women. The effects of group and the group × sex interaction were not significant.

No statistically significant differences were found between groups in thoracic kyphosis angle values, which averaged 49.7°, 49.8°, and 51.5° in men in the TBP, TNBP, and PAC groups, respectively, and 50.5°, 51.3°, and 46.5° in women. Similarly, lumbar lordosis depth (fleche lombaire) was 43.2 mm, 41.4 mm, and 44.6 mm in men and 44.5 mm, 45.8 mm, and 46.2 mm in women (*p* > 0.05). The effects of group, sex, and the group × sex interaction were not statistically significant.

No statistically significant differences were observed between groups in cervical lordosis depth (fleche cervicale) (*p* > 0.05). Mean values for men in the TBP, TNBP, and PAC groups were 69.9 mm, 68.6 mm, and 67.3 mm, respectively; for women, they were 54.4 mm, 51.4 mm, and 48.3 mm. A statistically significant effect was found for sex (F_1,100_ = 35.53, *p* < 0.001, η^2^ = 0.262), with higher values observed in men. The effect of the group and the group × sex interaction was not significant.

## 4. Discussion

The main finding of this study is that, in this sample, the analyses did not reveal any clear and consistent between-group differences across most static pelvic and spinal posture parameters. Three selective between-group differences were observed, namely pelvic rotation among men, coronal imbalance among women, and maximum right vertebral rotation among men. These findings suggest that any between-group differences, if present, may be selective, small in magnitude, and of uncertain clinical relevance rather than indicative of a single postural pattern clearly associated with low back pain. Moreover, relaxed standing posture may have limited value as an isolated marker of low back pain in amateur tennis players.

Similar results, i.e., no significant differences in posture between individuals with and without LBP, have been reported in adults who do not practice any sport [17,18,19].

An important interpretive consideration is that the present study assessed relaxed standing posture rather than dynamic spinal loading, sport-specific movement patterns, or functional postural control during tennis-related tasks. Therefore, the absence of consistent differences in static posture does not exclude the possibility that LBP in amateur tennis players is more closely related to movement-related, load-dependent, or technique-specific factors than to standing alignment alone.

In addition, all participants were assessed under pain-free conditions on the day of examination. Although this reduced the influence of acute pain-avoidance mechanisms on habitual standing posture, it may also have limited the ability to capture posture adaptations associated with symptomatic states.

In the present study, isolated significant differences were observed, including pelvic alignment in the transverse plane. Men in the TBP group presented a slight left pelvic rotation in relaxed standing (with the pubic symphysis oriented to the left). In contrast, the TNBP group showed a tendency toward right-sided rotation. Although the observed difference was small, it may reflect different postural control strategies or long-term adaptations resulting from specific training loads. However, the interpretation of this finding should be cautious, particularly given the lack of significant differences across most of the parameters analysed.

Women in the TBP group demonstrated significantly greater coronal imbalance compared to women in the PAC group. Roman et al. [15] indicated that this parameter is a factor associated with LBP in women. The mean value reported by Roman et al. was lower than that observed in women in the TBP group in the present study. This finding may reflect a local postural difference; however, its clinical meaning remains uncertain given the predominantly non-significant results across the remaining parameters. In the literature, results are inconsistent, with some studies showing no association [29] and others confirming such a relationship [30].

Greater right-sided spinal rotation in PAC men compared to those in the TBP group may indicate that practicing tennis, an asymmetric sport requiring substantial spinal rotational range of motion [31], does not lead to posture changes that cause LBP. Similar conclusions were drawn by Muyor et al. [32], who reported that tennis practice does not affect lumbar lordosis or thoracic kyphosis values in relaxed standing postures. Given the asymmetrical demands of tennis, this result may reflect sport-specific adaptation rather than a pain-related postural deviation; however, its clinical significance remains uncertain and should be interpreted cautiously.

One of the posture parameters associated with LBP is the lumbar lordosis angle [14,33]. In the present sample, we did not detect a clear between-group difference in lumbar lordosis angle. However, meta-analyses by Sadler et al. [33] and Chun [14] indicated that decreased lumbar lordosis was significantly associated with the development of LBP. In contrast, Khoshroo et al. [34] reported that increased lumbar lordosis in individuals older than 25 years who spend prolonged periods standing was associated with LBP. Due to conflicting findings among authors, sagittal spinal curvature morphology cannot be unequivocally identified as a risk factor for LBP.

Postural control is not merely a position but a complex pattern of reflexes, behaviors, and adaptive responses that maintain upright posture, which results from the coordinated activity of multiple muscles responsible for stability [35]. The present results suggest that static posture alone may not adequately explain the occurrence of LBP in amateur tennis players. Low back pain is likely to be multifactorial in this population, with potential contributions from training load, movement patterns, lumbopelvic function, sport-specific biomechanics, and prior musculoskeletal history. The lack of clear relationships suggests that associations between static posture and low back pain are complex and multifactorial, extending beyond simple morphological relationships.

An additional point to consider when interpreting the present findings is the operational definition of the TBP group. Because group allocation was based on self-reported tennis-limiting low back pain rather than specialist-confirmed diagnosis or clinical subclassification, heterogeneity within the symptomatic group cannot be excluded. This may have reduced the likelihood of identifying a consistent static postural pattern associated with low back pain. Only a comprehensive, multidimensional research approach can enable a more precise determination of the potential role of postural parameters in the pathomechanism of LBP in this population.

The present findings are broadly consistent with previous reports suggesting that practicing an asymmetric sport that requires a large range of spinal rotation [31] does not necessarily lead to permanent postural changes associated with LBP [32].

### Strengths and Limitations

This study has several strengths, including the use of a standardized radiation-free rasterstereographic method (DIERS Formetric 4D system) and the inclusion of both symptomatic and asymptomatic tennis players as well as physically active controls. The study contributes to the limited literature on long-term amateur tennis participation and selected spinal and pelvic alignment characteristics in relation to LBP.

However, several limitations should be considered. Because of the cross-sectional design, the findings should be interpreted as associations rather than causal relationships. Recruitment was volunteer-based, and key eligibility data on tennis participation, physical activity, and history of low back pain were self-reported, which may have introduced selection and recall bias. Although the final sample exceeded the a priori minimum for the main comparisons, the study may have been less sensitive to smaller effects, particularly in sex-stratified analyses, which should therefore be regarded as exploratory. In addition, the TBP group was defined by self-reported tennis-limiting low back pain rather than a specialist-confirmed diagnosis, so some clinical heterogeneity cannot be ruled out. The study also focused exclusively on static posture in relaxed standing and did not assess dynamic spinal loading, sport-specific movement patterns, or functional biomechanics during tennis activity, which limits ecological validity. Participants were examined under pain-free conditions on the day of assessment; although this improved standardization, it may have reduced the ability to detect posture adaptations associated with symptomatic states. Furthermore, the physically active control group was not formally matched to the tennis groups in terms of training load or activity type, and potentially relevant factors such as training load, dominant side, previous minor injuries, and technical characteristics of play were not fully controlled for. Finally, the findings apply to adult amateur tennis players and should be generalized to other populations with caution. Because numerous posture parameters were analyzed, isolated significant findings should be interpreted with caution, particularly given their uncertain clinical relevance.

## 5. Conclusions

In this cross-sectional sample of amateur tennis players, static pelvic and spinal posture parameters showed limited and inconsistent associations with low back pain. The observed between-group differences were selective and of uncertain clinical relevance, and the analyses did not reveal a consistent postural pattern distinguishing tennis players with low back pain from the comparison groups. These findings suggest that relaxed standing posture alone may have limited value for explaining low back pain in this population. Given the exploratory nature of some subgroup analyses and the symptom-based definition of the low back pain group, smaller or more heterogeneous effects cannot be excluded. Further multidimensional studies incorporating dynamic biomechanics, training-related factors, functional aspects of postural control, and clinically stratified characteristics of low back pain are warranted to clarify the mechanisms underlying low back pain in amateur tennis players.

## Figures and Tables

**Table 1:** Characteristics of the study population (mean ± SD, [min–max]).

Characteristic	TBP (n = 41)	TNBP (n = 48)	PAC (n = 27)
**Gender**	M = 26	M = 23	M = 15
	F = 15	F = 25	F = 12
**Age [years]**	M: 35 ± 8.6 [19–47]	M: 33 ± 9.4 [18–50]	M: 29 ± 8.7 [23–48]
	F: 37 ± 8.5 [23–50]	F: 36 ± 8.4 [23–50]	F: 31 ± 8.2 [22–49]
**Body mass [kg]**	M: 84 ± 9.2 [68–112]	M: 83 ± 12.1 [63–115]	M: 80.0 ± 11.4 [64–103]
	F: 69 ± 13.0 [50–98]	F: 67 ± 10.2 [54–88]	F: 58 ± 4.3 [52–65]
**Body height [cm]**	M: 182 ± 5.9 [172–197]	M: 182 ± 7.3 [171–196]	M: 180 ± 6.8 [166–194]
	F: 169 ± 7.6 [153–180]	F: 167 ± 8.0 [150–179]	F: 170 ± 6.4 [163–183]
**BMI [kg/m^2^]**	M: 25.6 ± 2.9 [21–33]	M: 25.1 ± 3.5 [21–36]	M: 24.5 ± 2.5 [20–30]
	F: 23.9 ± 4.4 [19–37]	F: 24.0 ± 3.5 [19–33]	F: 20.1 ± 1.1 [17–22]

TBP—Tennis Back Pain; TNBP—Tennis no Back Pain; PAC—Physically Active Control; M—Males; F—Females.

**Table 2:** Comparison of the study groups in terms of body posture parameters—pelvis.

Factor	Mean ± SD [Min–Max]		
	TBP	TNBP	PAC
**Pelvic tilt [mm]**	M: −2.15 ± 5.65 [−10–10]	M: −0.57 ± 3.90 [−9–6]	M: −2.20 ± 4.64 [−10–4]
	F: 0.88 ± 4.17 [−8–9]	F: −0.50 ± 4.67 [−10–4]	F: 0.25 ± 3.65 [−7–7]
**Pelvic rotation [°]**	**^ M: −1.12 ± 2.65 [−5–4]**	**^ M: 1.17 ± 2.44 [−3–6]**	M: 1.00 ± 2.97 [−4–6]
	F: 2.31 ± 2.23 [−2–6]	F: 0.93 ± 3.01 [−5–4]	F: 0.42 ± 2.53 [−4–6]
**Pelvic torsion [°]**	M: 0.35 ± 3.20 [−5–5]	M: −0.09 ± 3.06 [−7–5]	M: 1.67 ± 3.59 [−5–8]
	F: −0.69 ± 3.48 [−8–7]	F: −0.29 ± 3.06 [−7–4]	F: −0.92 ± 2.33 [−5–3]
**Coronal imbalance [mm]**	M: 6.81 ± 5.26 [−3–20]	M: 5.74 ± 10.58 [−11–26]	M: 6.20 ± 7.48 [−5–19]
	* F: −7.06 ± 5.71 [−15–5]	F: −5.29 ± 5.74 [−15–10]	* F: −0.58 ± 5.33 [−12–7]

TBP—Tennis Back Pain; TNBP—Tennis No Back Pain; PAC—Physically Active Control; M—Males; F—Females; ^—statistically significant differences between men’s groups (*p* < 0.05); *—statistically significant differences between women’s groups (*p* < 0.05).

**Table 3:** Comparison of the study groups in terms of body posture parameters—spine.

Factor	Mean ± SD [Min–Max]		
	TBP	TNBP	PAC
**Lumbar lordosis angle [°]**	M: 39.77 ± 7.83 [28–56]	M: 35.87 ± 7.22 [22–50]	* M: 34.80 ± 6.69 * [24–46]
	F: 46.69 ± 8.92 [30–73]	F: 48.43 ± 7.31 [33–58]	* F: 48.25 ± 6.15 * [38–60]
**Thoracic kyphosis angle [°]**	M: 49.73 ± 8.43 [33–69]	M: 49.78 ± 6.59 [39–72]	M: 51.47 ± 7.54 [38–66]
	F: 50.50 ± 9.62 [30–68]	F: 51.29 ± 11.15 [29–66]	F: 46.50 ± 8.70 [28–62]
**Fleche cervicale [mm]**	M: 69.85 ± 12.99 [48–97]	M: 68.61 ± 13.82 [44–100]	* M: 67.33 ± 11.4 * [43–80]
	F: 54.44 ± 15.58 [34–100]	F: 51.36 ± 15.29 [28–80]	* F: 48.25 ± 15.2 * [18–73]
**Fleche lombaire [mm]**	M: 43.19 ± 16.29 [16–70]	M: 41.43 ± 11.05 [25–65]	M: 44.60 ± 10.88 [19–63]
	F: 44.49 ± 12.97 [19–66]	F: 45.79 ± 13.15 [20–63]	F: 46.42 ± 11.19 [31–65]
**Trunk torsion [°]**	M: 1.85 ± 3.56 [−2–8]	M: 0.96 ± 3.25 [−2–9]	M: −0.07 ± 2.35 [−2–5]
	F: 1.88 ± 3.24 [−4–7]	F: 0.86 ± 2.67 [−2–5]	F: 0.08 ± 2.72 [−3–4]
**Apical deviation +max [mm]** **(maximum deviation to the right)**	M: 7.38 ± 4.29 [0–15]	M: 8.83 ± 5.87 [1–25]	M: 5.33 ± 4.11 [0–14]
	F: 9.69 ± 6.60 [1–26]	F: 10.36 ± 6.19 [2–22]	F: 7.67 ± 4.27 [3–16]
**Apical deviation -max [mm]** **(maximum deviation to the left)**	M: 2.54 ± 3.24 [0–12]	M: 1.04 ± 2.51 [−7–6]	M: 2.93 ± 2.67 [0–9]
	F: 2.88 ± 3.53 [0–9]	F: 1.71 ± 1.83 [0–5]	F: 2.17 ± 3.02 [0–10]
**Apical deviation RMS [mm]** **(spinal deviation RMS)**	M: 4.35 ± 2.42 [0–9]	M: 4.74 ± 2.77 [2–12]	M: 3.67 ± 1.81 [1–8]
	F: 5.88 ± 3.79 [1–17]	F: 5.50 ± 3.38 [0–12]	F: 4.50 ± 2.25 [2–9]
**Maximum vertebral rotation to the right [°]**	* M: 1.27 ± 2.01 [0–8] *	M: 2.30 ± 2.42 [0–8]	* M: 2.87 ± 1.78 [0–6] *
	F: 3.44 ± 2.65 [0–8]	F: 3.36 ± 2.94 [0–10]	F: 2.92 ± 3.01 [0–11]
**Maximum vertebral rotation to the left [°]**	M: 6.85 ± 3.83 [2–14]	M: 5.00 ± 3.40 [0–14]	M: 4.73 ± 3.19 [1–11]
	F: 4.50 ± 4.58 [0–16]	F: 5.50 ± 3.79 [0–12]	F: 5.00 ± 2.08 [2–9]
**Vertebral rotation RMS [°]**	M: 3.42 ± 1.88 [1–7]	M: 2.91 ± 1.32 [1–6]	M: 2.60 ± 1.31 [1–6]
	F: 3.25 ± 1.82 [1–9]	F: 3.29 ± 1.22 [1–6]	F: 2.67 ± 1.11 [1–5]
**Scoliosis angle [°]**	M: −3.19 ± 7.43 [−13–13]	M: −1.91 ± 7.91 [−17–13]	M: −1.93 ± 6.96 [−14–9]
	F: −7.81 ± 8.45 [−19–11]	F: −5.71 ± 8.16 [−14–14]	F: −2.33 ± 7.97 [−14–11]

TBP—Tennis Back Pain; TNBP—Tennis no Back Pain; PAC—Physically Active Control; M—Males; F—Females; *—significant effect was found for sex (*p* < 0.05).

## Data Availability

The results and raw dataset supporting the findings of this study are available from the corresponding author upon reasonable request via email.

## References

[1] Pluim B.M., Groppel J.L., Miley D., Crespo M., Turner M.S. (2018). Health benefits of tennis. Br. J. Sports Med..

[2] Kovacs M., Pluim B., Groppel J.L., Crespo M., Roetert E.P. (2016). Health, Wellness and Cognitive Performance Benefits of Tennis. J. Med. Sci. Tennis.

[3] Fu M.C., Ellenbecker T.S., Renstrom P.A., Windler G.S., Dines D.M. (2018). Epidemiology of injuries in tennis players. Curr. Rev. Musculoskelet. Med..

[4] Abrams G.D., Renstrom P.A., Safran M.R. (2012). Epidemiology of musculoskeletal injury in the tennis player. Br. J. Sports Med..

[5] Fiani B., Jarrah R., Wong A., Alamah A., Runnels J. (2020). Repetitive Traumatic Discopathy in the Modern-Era Tennis Player. Cureus.

[6] Fleisig G., Nicholls R., Elliott B., Escamilla R. (2003). Kinematics used by world class tennis players to produce high-velocity serves. Sports Biomech..

[7] Campbell A., Straker L., O’Sullivan P., Elliott B., Reid M. (2013). Lumbar Loading in the Elite Adolescent Tennis Serve: Link to Low Back Pain. Med. Sci. Sports Exerc..

[8] Dines J.S., Bedi A., Williams P.N., Dodson C.C., Ellenbecker T.S., Altchek D.W., Windler G., Dines D.M. (2015). Tennis Injuries: Epidemiology, Pathophysiology, and Treatment. J. Am. Acad. Orthop. Surg..

[9] Campbell A., O’Sullivan P., Straker L., Elliott B., Reid M. (2014). Back pain in tennis players: A link with lumbar serve kinematics and range of motion. Med. Sci. Sports Exerc..

[10] Johansson F., Gabbett T., Svedmark P., Skillgate E. (2022). External Training Load and the Association With Back Pain in Competitive Adolescent Tennis Players: Results From the SMASH Cohort Study. Sports Health.

[11] Zemková E., Kováčiková Z., Zapletalová L. (2020). Is There a Relationship Between Workload and Occurrence of Back Pain and Back Injuries in Athletes?. Front. Physiol..

[12] Errabity A., Calmels P., Han W.S., Bonnaire R., Pannetier R., Convert R., Molimard J. (2023). The effect of low back pain on spine kinematics: A systematic review and meta-analysis. Clin. Biomech..

[13] Sugavanam T., Sannasi R., Anand P.A., Ashwin Javia P. (2025). Postural asymmetry in low back pain—A systematic review and meta-analysis of observational studies. Disabil. Rehabil..

[14] Chun S.W., Lim C.Y., Kim K., Hwang J., Chung S.G. (2017). The relationships between low back pain and lumbar lordosis: A systematic review and meta-analysis. Spine J..

[15] Roman I., Luyten M., Croonenborghs H., Lason G., Peeters L., Byttebier G., Comhaire F. (2019). Relating the Diers formetric measurements with the subjective severity of acute and chronic low back pain. Med. Hypotheses.

[16] Yu Q., Huang H., Zhang Z., Hu X., Li W., Li L., Chen M., Liang Z., Lo W.L.A., Wang C. (2020). The association between pelvic asymmetry and non-specific chronic low back pain as assessed by the global postural system. BMC Musculoskelet. Disord..

[17] Kripa S., Kaur H. (2021). Identifying relations between posture and pain in lower back pain patients: A narrative review. Bull. Fac. Phys. Ther..

[18] Laird R.A., Gilbert J., Kent P., Keating J.L. (2014). Comparing lumbo-pelvic kinematics in people with and without back pain: A systematic review and meta-analysis. BMC Musculoskelet. Disord..

[19] Plandowska M., Kędra A., Kędra P., Czaprowski D. (2022). Trunk Alignment in Physically Active Young Males with Low Back Pain. J. Clin. Med..

[20] Degenhardt B.F., Starks Z., Bhatia S. (2020). Reliability of the DIERS Formetric 4D Spine Shape Parameters in Adults without Postural Deformities. BioMed Res. Int..

[21] Degenhardt B., Starks Z., Bhatia S., Kelley-Franklin G. (2017). Appraisal of the DIERS method for calculating postural measurements: An observational study. Scoliosis Spinal Disord..

[22] Lason G., Peeters L., Vandenberghe K., Byttebier G., Comhaire F. (2015). Reassessing the accuracy and reproducibility of Diers formetric measurements in healthy volunteers. Int. J. Osteopath. Med..

[23] Schroeder J., Schaar H., Mattes K. (2013). Spinal alignment in low back pain patients and age-related side effects: A multivariate cross-sectional analysis of video rasterstereography back shape reconstruction data. Eur. Spine J..

[24] Pizol G.Z., Miyamoto G.C., Cabral C.M.N. (2024). Hip biomechanics in patients with low back pain, what do we know? A systematic review. BMC Musculoskelet. Disord..

[25] Wilczyński J., Cieślik M., Maszczyk A., Zwierzchowska A. (2022). The Importance of Posture and Body Composition for The Stability and Selected Motor Abilities of Professional Handball Players. J. Hum. Kinet..

[26] Barczyk-Pawelec K., Bańkosz Z., Derlich M. (2012). Body postures and asymmetries in frontal and transverse planes in the trunk area in table tennis players. Biol. Sport.

[27] Grabara M. (2020). Posture of adolescent volleyball players—A two-year study. Biomed. Hum. Kinet..

[28] Guidetti L., Bonavolontà V., Tito A., Reis V.M., Gallotta M.C., Baldari C. (2013). Intra- and interday reliability of spine rasterstereography. BioMed Res. Int..

[29] Özyürek S., Genç A., Kul Karaalï H., Algun Z.C. (2017). Three-dimensional evaluation of pelvic posture in adolescents with and without a history of low back pain. Turk. J. Med. Sci..

[30] Chaléat-Valayer E., Mac-Thiong J.M., Paquet J., Berthonnaud E., Siani F., Roussouly P. (2011). Sagittal spino-pelvic alignment in chronic low back pain. Eur. Spine J..

[31] Brito A.V., Fonseca P., Costa M.J., Cardoso R., Santos C.C., Fernandez-Fernandez J., Fernandes R.J. (2024). The Influence of Kinematics on Tennis Serve Speed: An In-Depth Analysis Using Xsens MVN Biomech Link Technology. Bioengineering.

[32] Muyor J.M., Sánchez-Sánchez E., Sanz-Rivas D., López-Miñarro P.A. (2013). Sagittal spinal morphology in highly trained adolescent tennis players. J. Sports Sci. Med..

[33] Sadler S.G., Spink M.J., Ho A., De Jonge X.J., Chuter V.H. (2017). Restriction in lateral bending range of motion, lumbar lordosis, and hamstring flexibility predicts the development of low back pain: A systematic review of prospective cohort studies. BMC Musculoskelet. Disord..

[34] Khoshroo F., Seidi F., Bayattork M., Moghadas-Tabrizi Y., Nelson-Wong E. (2023). Distinctive characteristics of prolonged standing low back pain developers’ and the associated risk factors: Systematic review and meta-analysis. Sci. Rep..

[35] Li L., Zhang S., Dobson J. (2019). The contribution of small and large sensory afferents to postural control in patients with peripheral neuropathy. J. Sport Health Sci..

